# Challenges and Advances in the Detection of Leukemic Blasts in Cerebrospinal Fluid in Pediatric Acute Lymphoblastic Leukemia

**DOI:** 10.3390/cancers18050840

**Published:** 2026-03-05

**Authors:** Zhongbo Hu, Shuyu E

**Affiliations:** 1 Pediatric Hematology and Oncology, Haley Center for Children’s Cancer and Blood Disorders, Orlando Health Arnold Palmer Hospital for Children, Orlando, FL 32806, USA; 2 Pediatrics, Department of Clinical Sciences, College of Medicine, Florida State University, Orlando Regional Campus, Orlando, FL 32801, USA; 3 Department of Pathology, St. Jude Children’s Research Hospital, Memphis, TN 38105, USA; shuyu.e@stjude.org

**Keywords:** central nervous system leukemia, cytomorphology, terminal deoxynucleotidyl transferase, flow cytometry, qPCR, ddPCR, next generation sequencing

## Abstract

Central nervous system (CNS) evaluation for leukemic involvement is essential both at initial diagnosis and throughout relapse surveillance in childhood acute lymphoblastic leukemia (ALL). Accurate CNS risk classification is a cornerstone of individualized chemotherapy and has significantly advanced treatment strategies. However, detecting leukemic cells in the cerebrospinal fluid (CSF) is challenging, particularly when only a small number of cells are present. While cytomorphology remains a standard diagnostic method, it is limited by low sensitivity and interobserver variability, especially in low-cellularity or equivocal samples. Flow cytometry offers superior sensitivity and specificity and is increasingly recommended to confirm or clarify ambiguous findings. Current guidelines support the use of both cytomorphologic review and flow cytometry to maximize diagnostic accuracy. Evidence consistently demonstrates that any detectable CSF blasts—even in the setting of low WBC counts—are associated with increased risk of CNS relapse and poorer outcomes, underscoring the importance of risk-adapted CNS-directed therapy. Although the prognostic significance of isolated flow-only positivity remains under study, emerging data suggest that timely therapeutic intensification may mitigate adverse outcomes. Additional modalities, including advanced flow cytometry and molecular assays, may further refine CSF assessment in the future. This review summarizes current diagnostic approaches and highlights the need for standardized protocols for CSF evaluation in pediatric ALL.

## Simple Summary

Checking whether leukemia has spread to the brain is a key part of caring for children with acute lymphoblastic leukemia. When leukemia cells are found in the fluid surrounding the brain and spinal cord, the disease is harder to treat and the risk of relapse is higher, requiring more intensive therapy. Even very small numbers of leukemia cells in this fluid can increase the risk of leukemia coming back. Advances in treatments designed to protect the brain and spinal cord have greatly improved survival for children with acute lymphoblastic leukemia. However, detecting rare leukemia cells remains challenging because only small amounts of fluid and low numbers of cells are available for testing. This review describes current available methods used to identify leukemia cells in brain and spinal cord fluid, highlighting their importance, strengths, and limitations in guiding treatment and preventing relapses.

## 1. Introduction

Central nervous system (CNS) leukemia is relatively uncommon, occurring in fewer than 5% in pediatric acute lymphoblastic leukemia (ALL) cases at initial presentation and in approximately 5–10% of relapsed cases [1]. CNS evaluation for leukemic involvement is crucial both at initial diagnosis and throughout relapse surveillance in childhood ALL. Accurate CNS risk classification plays a pivotal role in guiding individualized chemotherapy and has substantially improved treatment outcomes. CNS involvement at diagnosis is considered a high-risk feature and presents a significant therapeutic challenge. During treatment and post-therapy follow-up, CNS relapses remain a major concern because it is associated with poor prognosis and requires vigilant monitoring. Notably, the development and refinement of CNS-directed therapy represent one of the most significant advances in the treatment of leukemia over the past century [2,3,4,5].

## 2. CNS Leukemia

The definition of CNS leukemia has evolved over the past three decades. It was initially described as pleocytosis accompanied by neurologic symptoms. The criteria later expanded to include a cerebrospinal fluid (CSF) white blood cell (WBC) count greater than 5 WBCs/µL with unequivocal lymphoblasts identified on cytocentrifuge [6]. Currently, CNS leukemia is defined as the presence of leukemia cells in the CSF or leukemic involvement of the CNS manifested as meningeal infiltration, intravascular aggregates, or tumor masses [7,8]. Clinically affected patients may present with neurological symptoms, such as cranial nerve palsies, headache, nausea, vomiting, seizures, or altered mental status.

CNS involvement is classified using standardized CNS staging, which incorporates CSF WBC counts and the morphological identification of blasts on cytocentrifuged preparations. Patients are categorized as CNS1, CNS2, or CNS3 status based on these criteria [9,10]. Table 1 highlights two major CNS classification systems that are currently used by European/St. Jude Children’s Research Hospital and Children’s Oncology Group (COG) [9,10]. Some institutions, including St. Jude Children’s Research Hospital and the National Comprehensive Cancer Network (NCCN), further recommend terminal deoxynucleotidyl transferase (TdT) staining and deep sequencing in patients with fewer than 5 WBCs/µL in the CSF but morphologically identifiable blasts [11].

The presence of blasts in CSF—even when WBC counts are low (CNS2)—is associated with a significantly increased risk of CNS relapse and inferior event-free survival. A landmark study by Pui and colleagues in 1993 demonstrated that patients with <5 WBCs/µL but detectable blasts had worse five-year survival compared to those without blasts, and that CSF blasts in this range were an independent risk factor of isolated CNS relapse [4]. More recent large cohort studies have confirmed that any detectable CSF blasts, regardless of WBC count, increase the risk of CNS relapses and worsen long-term outcomes. These findings underscore the need for risk-adapted CNS-directed intensification in affected patients [10,12]. Additionally, traumatic lumbar punctures, which are commonly defined as CSF containing at least 10 red blood cells (RBCs) per µL, with blast present are well documented as another risk factor for CNS relapse [3,13]. Because early detection of rare CSF blasts is essential for preventing relapse and optimizing therapy, sensitive and reliable diagnostic methods are critical [3].

A major challenge for hematopathologists is that CSF samples often contain very few leukemic cells, making accurate diagnosis difficult. This review summarizes currently available methods for detecting leukemia cells in CSF, highlighting the strengths and limitations of each diagnostic modality.

## 3. Biology of CSF

Normal CSF is a clear, watery fluid that cushions the brain and spinal cord. It typically contains 0–5 WBCs/µL, most of which are lymphocytes (65–80%) and monocytes (10–30%), and RBCs should not be present. An amount of 0.5 mL of normal CSF will yield ~30–50 cells by cytocentrifugation. Rare squamous epithelial cells or talcum particles may appear as contaminants from skin or gloves [14]. Opening pressure in children is generally less than 28 cm H_2_O (upper limit by 90th percentile) for most children when lying down [15,16,17]. In children aged 1–96 months, the median opening pressure is about 19.2 cm H_2_O (10th–90th percentile: 8–28.9 cm H_2_O), while in those older than 96 months, the median is 22 cm H_2_O (10th–90th percentile: 11.8–35 cm H_2_O) [18].

## 4. Cytomorphology

Cytomorphology assessment of cytospin slides remains the historical gold standard for detecting CNS leukemia due to its high specificity [19]. However, this method has several notable limitations, including low sensitivity, subjectivity, and reduced reproducibility—particularly when leukemia cells are sparse or morphologically ambiguous in the CSF. Historically, approximately 45% of patients with acute leukemia and suspected CNS involvement have negative CSF morphology [20]. This paradigm may be subject to future refinement as more sensitive and objective diagnostic modalities are developed and validated.

**Figure 1:**
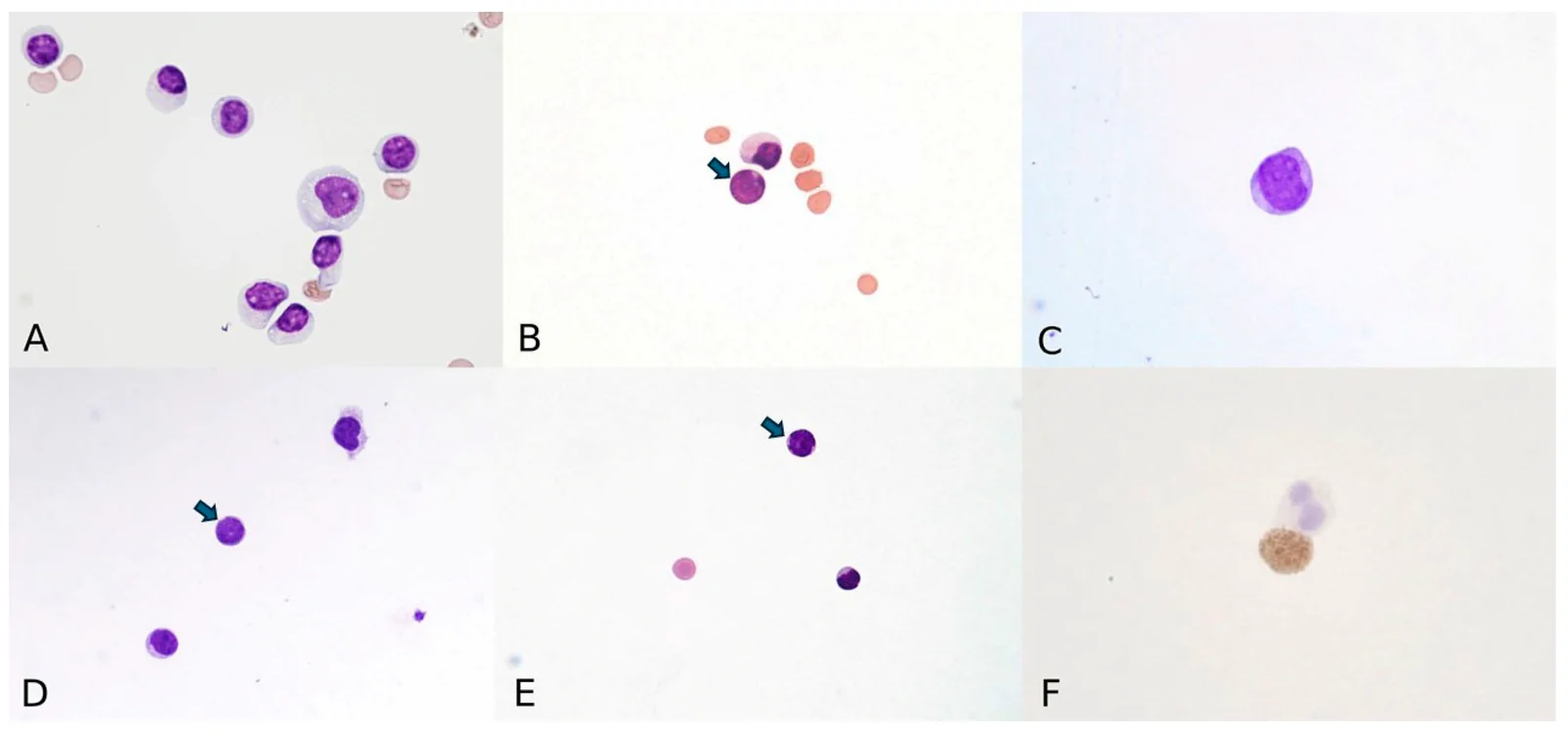
CSF cytomorphology (magnification 1000× oil). (**A**) Reactive pleocytosis with normal lymphocytes. (**B**) Arrow shows a pre-B ALL blast. (**C**–**E**) Lymphocytes post chemotherapy. Arrows (**D**,**E**) show lymphocyte changes. (**F**) A TdT-positive lymphoblast.

The 2017 College of American Pathologists and American Society of Hematology recommend that all CSF samples in ALL undergo both cells count and cytomorphologic review [21]. However, they also acknowledge the limitations of cytomorphology, particularly in cases with low cell counts or equivocal findings [21]. The standard process involves collecting CSF 1–3 mL, preparing a smear via cytocentrifugation, and examining the smear microscopically for the presence of leukemia blasts. Accurate interpretation requires prompt sample processing within 1–2 h, appropriate staining, and expert morphological assessment [22]. The May–Grunwald–Giemsa stain is commonly used to enhance cellular detail and differentiate cell types. Sometimes, a modified quicker-turnaround one-step Wright–Giemsa staining method is used [23]. Pathologists typically identify leukemic blasts by their slightly larger size than small mature lymphocytes, increased nuclear-to-cytoplasmic ratio, smooth and finely dispersed chromatin, smooth to notched nuclear contours, and prominent nucleoli [24] (Figure 1A,B). The scant cytoplasm is homogeneously basophilic and agranular but may show occasional small pale vacuoles and/or fine granules. Many mitoses can be observed [14].

Despite these efforts, cytomorphology remains a subjective method with low sensitivity and limited reproducibility, particularly when leukemic cells are present in low numbers. The literature consistently indicates that conventional cytospin cytomorphology has a sensitivity of less than 50% for detecting CSF leukemia and is generally able to identify malignant cells only when they constitute approximately 5% or more of the total cell population (i.e., a detection threshold of ~5 × 10^−2^, or 1 in 20 cells) [25]. Other studies have reported that CSF cytology failed to demonstrate lymphoma or leukemia cells in 37.5% of all cases [19]. During chemotherapy, morphological changes due to treatment effects or intercurrent infections may further complicate interpretation. Intrathecal chemotherapy can generate inflammation and activate lymphocytes. Activated lymphocytes may be morphologically atypical and can be misdiagnosed as blasts [19] (Figure 1C). Chemotherapy and associated immunosuppression can induce cytologic alterations, such as cellular atypia, reactive changes, or increased background debris, which may mimic or obscure malignant cells (Figure 1D,E). Additionally, opportunistic infections can cause pleocytosis and cytologic atypia, further confounding the distinction between leukemic infiltration and infectious or reactive processes [26,27]. These cytologic alterations can lead to diagnostic uncertainty and may contribute to false-negative or false-positive assessments of CNS leukemia involvement [25,28,29,30].

Technical limitations also contribute to diagnostic challenges, including small sample volumes, rapid cell degeneration due to the low nutrient environment of CSF, and the dependence on immediate sample processing [19]. Studies have shown that an optimal CSF volume of approximately 8 mL is recommended to ensure an adequate specimen for evaluation. In pediatric cases, where obtaining this volume may be challenging, a minimum volume of 1–2 mL is considered acceptable [22]. Collectively, these factors limit the sensitivity of morphology alone.

## 5. Immunocytochemistry and TdT Staining

Immunocytochemistry can improve the specificity of cytomorphology but does not significantly increase sensitivity, especially when the sample contains <10 WBCs/µL or only a small proportion of blasts. Various immunoperoxidase and immunoalkaline phosphatase methods have been adapted for CSF evaluation, with alkaline phosphatase–antialkaline phosphatase preferred for low-cellularity smears [31]. However, identifying a leukemia-specific antigen suitable for routine CNS assessment remains challenging. Common acute lymphoblastic leukemia markers were thought to be a good fit for diagnosis of CNS leukemia [32]. Earlier use of CALLA (CD10) has largely been abandoned due to false-positive staining in non-malignant lymphocytes [33,34]. Currently, these routine immunocytochemistry staining methods are used less frequently in clinical practice. However, many institutions continue to utilize TdT staining because of its relatively high specificity and sensitivity.

### TdT

TdT is an enzyme found in lymphoid precursors in the thymus and bone marrow, and its presence in CSF indicates the presence of leukemic blasts, aiding in the diagnosis and management of CNS leukemia. TdT staining is a valuable tool and specific immunochemistry staining method in diagnosing and classifying ALL, particularly in CSF samples, where it helps identify lymphoblasts. It can help distinguish normal lymphocytes from leukemic cells in questionable conditions and differentiate ALL from other types of leukemia, particularly acute myeloid leukemia (AML) [35]. Typically, TdT has low expression in AML. TdT is present in the nucleus and positive immunohistochemical stain shows a nuclear pattern (Figure 1F). TdT staining can detect leukemic blasts in CSF even when they are not visible by standard cytomorphology, which can provide early detection.

TdT staining is more sensitive than cytomorphologic methods for detecting low levels of lymphoblasts in CSF. In a landmark 5-year prospective study of 113 children with TdT-positive malignancies, a TdT immunofluorescence assay detected CNS involvement in 85 samples (5.2%) that were negative or equivocal by conventional cytomorphology [35]. Multiple studies have confirmed the superior diagnostic accuracy of combining TdT with cytomorphology. In one study evaluating 60 CSF samples from 28 children with TdT-positive ALL, concordance between cytomorphology and TdT was observed in 55 of 60 samples; notably, 72% of TdT-positive samples were obtained from children with CSF cell counts <10 WBCs/µL [36]. Another study demonstrated that conventional cytology alone correctly identified only 64% of cases (using TdT as the reference standard), whereas high-power microscopy increased correlation to 82%, and the combined use of cytology with TdT knowledge improved correlation to 85% [37]. The turnaround time (TAT) of TdT staining is typically the same day.

By increasing sensitivity, TdT staining helps reduce the risk of missing CNS involvement in ALL. While TdT is a strong marker for ALL, it is not entirely specific, as TdT expression can be seen in other conditions, such as some types of AML and other lymphoid neoplasms [38]. Proper interpretation of TdT staining results requires expertise in hematopathology. As mentioned in the introduction, some institutions, including St. Jude Children’s Research Hospital, have been using TdT staining in patients with fewer than 5 WBCs/µL in the CSF but morphologically identifiable blasts [6].

Current practice has evolved beyond standalone TdT staining. The American Society of Clinical Oncology guidelines note that TdT immunohistochemistry on cytospin preparations has historically been used for CNS disease detection in ALL; however, multicolor flow cytometry (6–10 colors) has been widely adopted in many centers, incorporating TdT as one of the core markers and providing greater sensitivity and specificity [21].

## 6. Flow Cytometry

Flow cytometry plays an increasingly important role in detecting CNS involvement in pediatric ALL, particularly in cases with low CSF cell counts or equivocal cytology. Both the College of American Pathologists and the American Society of Hematology acknowledge the limitations of morphology assessment alone and support the use of flow cytometry to clarify questionable findings [21]. By employing fluorescently labeled antibodies, flow cytometry enables sensitive immunophenotypic characterization and accurate detection of leukemic blasts.

Flow cytometry is an ancillary technique that analyzes light scattering and fluorescence emitted by individual cells as they pass through a laser beam in a fluid stream. CSF cells are stained with antibodies targeting specific surface antigens, allowing precise identification and classification of cell populations. The method offers rapid TAT, with most assays completed on the same day. It is considered highly reliable for detecting CNS involvement in pediatric ALL. In contrast, cytomorphology may be affected by multiple factors, including sample processing, patient age, temperature, staining techniques, timing of intrathecal therapy, and leukemia genetics. Consequently, many pathology laboratories routinely combine cytospin evaluation with CSF flow cytometry to enhance immunophenotypic characterization and definitive cell identification.

Since the 1980s, flow cytometry has been explored as a more sensitive alternative to conventional cytology for detecting leukemic cells in CSF [39], and accumulating evidence supports its superior sensitivity and specificity, particularly in clarifying ambiguous cases [9,27,40,41]. Multicolor flow cytometry can detect leukemia cells at levels as low as 0.2% of total CSF lymphocytes, corresponding to a sensitivity of 10^−3^ to 10^−4^ (i.e., one leukemia cell among 1000–10,000 normal cells). Its diagnostic yield has been reported to be more than twice that of conventional cytomorphology [3,30], consistently demonstrating higher sensitivity and specificity for identifying leukemic blasts in CSF and is increasingly supported for resolving diagnostically uncertain cases [40,41,42,43]. This high sensitivity is especially valuable when cytologic interpretation is confounded by treatment-related changes or infections [25,30].

Since 2001, multiple studies have compared cytology and flow cytometry for the detection of CNS leukemia [19,25,44]. Crespo-Solis et al. (2012) [19] summarized these data, demonstrating wide variability in concordance rates (37.5–94%) and highlighting the complementary role of flow cytometry (Table 2).

Beyond its diagnostic utility, flow cytometry provides important prognostic information. True CNS involvement is defined by detection of cells with abnormal immunophenotypes matching the known or suspected malignancy. Isolated flow cytometry positivity in the absence of morphologic confirmation (cytology-negative/flow-positive), often termed “flow-only positivity” or “occult CNS disease”, raises two key questions: how flow cytometry positivity should be defined and whether it carries clinical significance.

Traditionally, the recommended minimum event acquisition target for CSF flow cytometry refers to the minimum number of viable leukocytes required to ensure adequate analysis, which is distinct from the minimum number of abnormal events required to classify a sample as positive. The literature suggests that at least 100–220 viable leukocytes should be acquired to permit adequate flow cytometric evaluation of CSF samples [49]. In contrast, the diagnostic threshold for CNS leukemia involvement has traditionally been defined as ≥10 phenotypically abnormal events forming a cluster [22,33]. This threshold was established to balance sensitivity with specificity, acknowledging the technical challenges of CSF analysis, including low cellularity and potential contamination. Flow-only positivity has been associated with higher CNS relapse rates and inferior survival in several studies, warranting structured interpretation and management [33,50]. More recently, a lower threshold of ≥3 events has been validated to significantly improve sensitivity while maintaining prognostic relevance [51]. Patients with 3–9 abnormal events demonstrated worse event-free survival and higher cumulative incidence of CNS relapses, particularly in hyperdiploid ALL receiving less intensive CNS-directed therapy [51]. In contrast, borderline findings with 1–2 events represent the most challenging interpretive category and should be reported as indeterminate rather than definitively positive or negative, with recommendations for repeat sampling and clinical correlation [22,33]. Cases with 0 abnormal events are reported as negative by flow cytometry, even in the setting of high clinical suspicion.

Several technical factors may affect flow cytometry interpretation:

**Sample adequacy**: Optimal sensitivity ideally requires acquisition of ≥4 million cells, although CSF hypocellularity often limits this [52].

**Cluster formation**: Abnormal events must form a distinct cluster with an aberrant immunophenotype matching the diagnostic leukemia-associated immunophenotype [33].

**Cell viability**: Poor viability may result in false-negative findings. Samples should be processed within 24 h, and preservation media such as Transfix may be considered if delays are anticipated [33,44].

**Traumatic tap**: Peripheral blood contamination requires careful interpretation. Comparison of the CSF WBC/RBC ratio with the peripheral blood WBC/RBC ratio helps assess contamination [11].

The prognostic significance of flow-only positivity remains under investigation. Some evidence suggests that the associated adverse risk may be mitigated by intensified CNS-directed therapy [8,21,42]. Several studies have reported that patients with flow cytometry-only positivity who receive intensified therapy do not experience significantly worse event-free survival compared with patients without CNS involvement [8,21,42]. However, other studies have demonstrated that CSF involvement detected by flow cytometry is associated with increased recurrence risk [20]. Even low-level CSF blasts (e.g., CNS2 status) correlate with higher CNS relapse rate and inferior event-free survival. Large cohort studies have shown that any detectable CSF blasts, regardless of WBC count, are associated with poorer outcomes, supporting risk-adapted intensification of CNS-directed therapy [7,53,54].

Reflecting its diagnostic and prognostic value, NCCN recommends routine use of flow cytometry in combination with cytomorphology to improve detection CNS involvement in hematologic malignancies [55]. In line with the recommendations, many academic institutions have adopted this combined approach in routine clinical practice. Integration of cytomorphology and flow cytometry enhances diagnostic sensitivity and refines risk stratification.

Despite these advantages, several challenges remain. Current COG CNS classification thresholds were established based on cytospin morphology rather than flow cytometry. In addition, cells in paucicellular CSF samples undergo rapid degeneration, complicating flow cytometry analysis, which requires intact cells. Optimal performance depends on preserved cell surface markers, prompt sample processing—ideally within 1–2 h of collection—and the use of appropriate stabilization or cell preservation media [56,57].

Accurate detection requires optimal laboratory conditions [19]: CSF samples of at least >2 mL; timely data acquisition with adequate event counts within 2 h of sample collection, although samples collected in RPMI 1640 medium, which neutralizes the cytotoxic effects of CSF, may be stored at room temperature for up to 18 h; a properly calibrated flow cytometer; and 6–10 color immunofluorescent staining using monoclonal antibody core panels for diagnosis [21,58]. Core panels must accomplish two essential tasks: determine cell lineage and identify abnormal immunophenotypes distinguishing leukemic cells from normal lymphoid progenitors.

For B-cell ALL, backbone markers typically include CD19, CD10, and CD34, combined with markers selected based on the diagnostic immunophenotype from bone marrow [52,59]. The EuroFlow standardized approach incorporates CD19, CD10, and CD34 as consistent backbone markers, with additional markers such as CD20, CD38, CD45, and TdT forming the essential panel [52]. These panels enable identification of B-lymphoblasts in >98% of cases. For T-cell ALL, core markers include cytoplasmic CD3, CD5, CD7, and TdT or CD34, establishing T-lineage and immaturity [59]. Additional T-lineage markers (CD2, CD4, CD8) further characterize the immunophenotype [60], while extended markers may include CD1a (for thymic subtype), surface CD3 (for mature subtype), CD99, and myeloid markers (CD13, CD33) to identify early T-cell precursor phenotypes [59,60].

In equivocal cases with paucicelluar CSF samples, a volume of approximately 8–10 mL may improve detection [20,22]. In pediatric cases—where large volumes may be difficult to obtain—a volume of approximately 5–10 mL is generally considered optimal.

Emerging innovations, including dried antibody reagents and spectral flow cytometry capable of analyzing more than 30 fluorochrome-conjugated antibodies in a single tube, promise to further improve the diagnostic yield, particularly in low-cellularity specimens [20,61]. These advances may enhance both sensitivity and efficiency of CSF analysis, further refining CNS leukemia detection and risk stratification in pediatric ALL.

## 7. Molecular Techniques

### 7.1. Fluorescent In Situ Hybridization (FISH)

FISH can be used to detect specific chromosomal abnormalities or gene rearrangements in leukemic cells. Interphase FISH is a well-validated technique for identifying cytogenetic abnormalities and is routinely used to support the diagnosis of acute leukemia. When leukemia-associated cytogenetic alterations are known at initial diagnosis, FISH may be applied to CNS specimens to improve the sensitivity of CNS leukemia detection to 5% [62]. In one study, FISH performed on CSF cytospin samples identified an additional 13% of patients with CNS leukemia among 23 specimens in which cytologic interpretation was not possible due to low cellularity [7]. The TAT for FISH can be as fast as 24–48 h. A major disadvantage of FISH is its limited applicability, as only leukemias with available, disease-specific probes can be evaluated. A significantly streamlined FISH protocol has also been reported for detecting residual leukemic blasts in ALL using CSF as a complementary diagnostic specimen [63].

### 7.2. Polymerase Chain Reaction (PCR)

Analysis of rearranged immunoglobulin (*Ig*) genes in B lymphocytes and T-cell receptor (*TCR*) genes in T lymphocytes (DNA target) has served as a cornerstone for the diagnosis and monitoring of leukemia and lymphoma for more than three decades. PCR enables amplification of leukemia-specific DNA sequences from CSF, allowing highly sensitive detection of leukemia cells, particularly in the context of minimal residual disease (MRD) assessment [64,65,66,67].

The first application of PCR for detecting minimal leukemic cells in CSF was reported in the early to mid-1990s. A landmark study published in 1998 described the use of PCR to detect Vδ2Dδ3 rearrangements of the TCR gene in CSF samples for the diagnosis and monitoring CNS leukemia in children with ALL, demonstrating superior sensitivity compared with routine CSF examination methods [68]. Since then, multiple groups have explored a range of PCR-based approaches for identifying CSF involvement in leukemia and lymphoma, highlighting several advantages over cytology and flow cytometry.

First, PCR markedly increases sensitivity by amplifying trace amounts of DNA and does not require intact cells, which is particularly advantageous in paucicellular CSF samples. Second, several advanced PCR-based techniques have been developed to further improve both sensitivity and specificity. Although the TAT for PCR is typically 2–3 days, its improved analytical sensitivity makes it a valuable adjunct in CSF evaluation. Table 3 summarizes the advantages and limitations of these MRD detection methods for CSF leukemia detection in comparison with cytomorphology and flow cytometry [58,62,69,70].

#### 7.2.1. Real-Time/Quantitative PCR (qPCR)

qPCR is commonly used to detect *Ig* heavy-chain (*Ig*H) or *TCR* gene rearrangements to demonstrate clonality and can achieve a sensitivity of up to 10^−5^ (one leukemic cell among 100,000 normal lymphoid cells) [62]. qPCR is often used clinically alongside flow cytometry to enhance the detection of occult CNS involvement to refine risk stratification and diagnose relapses [65]. However, false-positive results may occur in inflammatory conditions due to restricted Ig gene rearrangements in reactive B lymphocytes. Additionally, this technique is not applicable to immature leukemias lacking *Ig*H or *TCR* rearrangements.

#### 7.2.2. Leukemia-Specific qPCR

By targeting known leukemia-specific fusion transcripts (RNA target) or clonal gene rearrangements (DNA target) identified at diagnosis, leukemic-specific qPCR can detect leukemic blasts in CSF with greater sensitivity than conventional cytomorphology [65,71]. However, this approach is not routinely implemented in clinical practice and is primarily used in research setting or select cases of MRD monitoring [67].

#### 7.2.3. Digital Droplet PCR (ddPCR)

ddPCR is a newer, highly sensitive quantitative technique—often referred to as a third-generation PCR method—that enables precise measurement of low-level leukemic DNA. In ddPCR, the PCR reaction mixture is partitioned into thousands of nanoliter-sized droplets, each containing zero, one, or a few copies of target DNA. PCR amplification occurs independently within each droplet, and fluorescence signals are measured at the end of the reaction [72]. Absolute quantification of target DNA is calculated based on the proportion of positive droplets using Poisson distribution [73].

Although the ddPCR technique remains in the clinical study phase, it is expected to be adopted into routine clinical practice in the near future due to its significant advantages.

The advantages of ddPCR include absolute quantification without the need for a standard curve, high tolerance to PCR inhibitors, detection of small fold changes (~10%), superior precision, and suitability for low-abundance targets. ddPCR can achieve a limit of quantification of approximately 10^−4^ and limit of detection approaching 10^−5^ in CSF leukemia detection [74] and has consistently outperformed qPCR in terms of quantitative limit of detection and sensitivity [70,75]. In particular, ddPCR reduces the proportion of low-level false-positive measurements and has shown promise for MRD evaluation in ALL cases with very low or negative qPCR results. One study reported that among slow early responders in pediatric ALL treatment, most relapses occurred in patients with quantifiable ddPCR MRD at day +78, whereas patients with negative or non-quantifiable ddPCR MRD at this time point demonstrated more favorable outcome [76]. Given its sensitivity, ddPCR holds particular promise for the detection of CNS leukemia in low-cellularity CSF samples. To date, studies applying ddPCR to CSF have primarily focused on microRNA biomarkers for pediatric leukemia CNS involvement [75]. Beyond microRNA, the literature demonstrates broader applications of ddPCR in CSF for detecting various molecular targets in pediatric malignancies. In pediatric ALL specifically, ddPCR has been used to detect patient-specific structural variants identified by whole-genome sequencing, successfully identifying MRD in CSF samples and detecting low-grade CNS involvement [74].

### 7.3. MicroRNA (miRNA)

miRNAs are small (21–23 nucleotides), single-stranded, non-coding RNAs that regulate gene expression through post-transcriptional mechanisms, including binding to complementary sequences in the 3′ untranslated region of messenger RNA (mRNA), resulting in mRNA degradation, poly(A) tail shortening, or translational repression [77]. miRNAs are released into the extracellular space via extracellular vesicles and are more resistant to RNases than mRNA. Owing to their stability and ability to circulate in body fluids, miRNAs have been investigated as liquid biopsy biomarkers in peripheral blood, plasma, serum, urine, and CSF [78].

Secreted miRNAs reflect the biological characteristics of their tissue of origin, and accumulating evidence suggests that they represent promising biomarkers for disease monitoring following therapy [79,80]. MiR-181a and MiR-181b-5p, for example, have been explored as potential MRD biomarkers for detecting CNS involvement in childhood ALL [81,82,83]. In these studies, both qPCR and ddPCR were used for miroRNAs detection. As discussed above, this modality remains under clinical investigation and has not yet been fully integrated into routine practice.

### 7.4. Next Generation Sequencing (NGS)

NGS is a high-throughput molecular technology that enables simultaneous sequencing of millions of DNA fragments in parallel. Following nucleic acid extraction and library preparation, amplified DNA fragments are sequenced and aligned to a reference genome or patient-specific clonotypic sequences to identify mutations or leukemic clones. Its deep sequencing capacity allows highly sensitive detection of low-frequency leukemic cells, making it particularly valuable for CNS disease monitoring. NGS-based MRD assessment using *Ig* and *TCR* gene rearrangements can achieve a sensitivity of 10^−6^ cells [60,62,84]. NGS is increasingly utilized as a high-sensitivity adjunctive tool for the detection and monitoring of CNS leukemia, particularly in cases where conventional methods, such as cytomorphology and flow cytometry, yield inconclusive results. Achievement of NGS-negative status in the CNS compartment has been strongly associated with superior event-free and overall survival. While still under clinical investigation, NGS is already being implemented in practice at several institutions [85]. However, application of NGS to CSF samples is limited primarily by the low biomass and limited sample volume typically available from the lumbar puncture. Additional barriers include limited availability in clinical laboratories and longer TAT—often ranging from 2 to 4 weeks—which may delay CSF risk stratification and therapeutic decision-making.

The use of cell-free DNA (cfDNA) shed into the CSF represents a promising strategy to overcome these limitations. CSF-derived cfDNA has been shown to harbor cancer-specific genomic alterations and may enable sensitive NGS-based monitoring for CNS disease burden [86]. Further validation is required before widespread clinical implementation.

To summarize, in contemporary practice, CSF evaluation for leukemia involvement relies on the coordinated integration of cytology, flow cytometry, and, selectively, molecular assays. Cytomorphology remains the universal first-line test and provides essential morphologic context; however, its limited sensitivity in low-cellularity or MRD settings necessitates adjunctive methods. Flow cytometry is routinely performed in many tertiary and pediatric centers at initial diagnosis, relapses, or other high-risk scenarios, where it functions as a parallel standard modality to enhance sensitivity and provide immunophenotypic confirmation. In lower-risk or surveillance settings, however, flow cytometry is often used in a confirmatory or reflex manner, triggered by equivocal cytology, atypical cells, or heightened clinical concern. Discordant results—particularly flow-positive/cytology-negative cases—are interpreted in conjunction with systemic disease status and may prompt repeat sampling or closer monitoring.

Molecular assays currently occupy a more targeted and stratified role. qPCR is used in routine practice when a known molecular target is available and is most incorporated into established MRD monitoring programs, with selective application to CSF specimens. ddPCR, offering greater analytical sensitivity, is increasingly implemented in specialized centers for ultra-low-level detection or adjudication of borderline cases. NGS remains largely confined to specialized or research settings for CSF evaluation because of cost, TAT, and technical considerations, although it plays an important role in systemic genomic profiling and complex relapse assessment. Collectively, these modalities function within a multimodal diagnostic algorithm in which cytology provides structural and morphologic context, flow cytometry enhances sensitivity and specificity, and molecular assays deliver highly sensitive, target-specific detection in selected clinical scenarios.

In real-world decision-making, these approaches are integrated according to clinical context. For example,

- At **initial leukemia diagnosis in high-risk patients**, cytology and flow cytometry are typically performed concurrently; molecular testing may be added when a known target is available.
- During **routine surveillance with low clinical suspicion**, cytology may be performed alone, with reflex flow cytometry if abnormalities are identified.
- In **discordant cases (e.g., flow-positive/cytology-negative)**, correlation with systemic MRD status and, when available, targeted molecular testing can help distinguish true CNS involvement from technical artifacts.
- In **MRD-driven treatment adaptation**, qPCR or ddPCR may provide additional sensitivity beyond morphology and flow cytometry, particularly in specialized centers.

## 8. Conclusions

**Figure 2:**
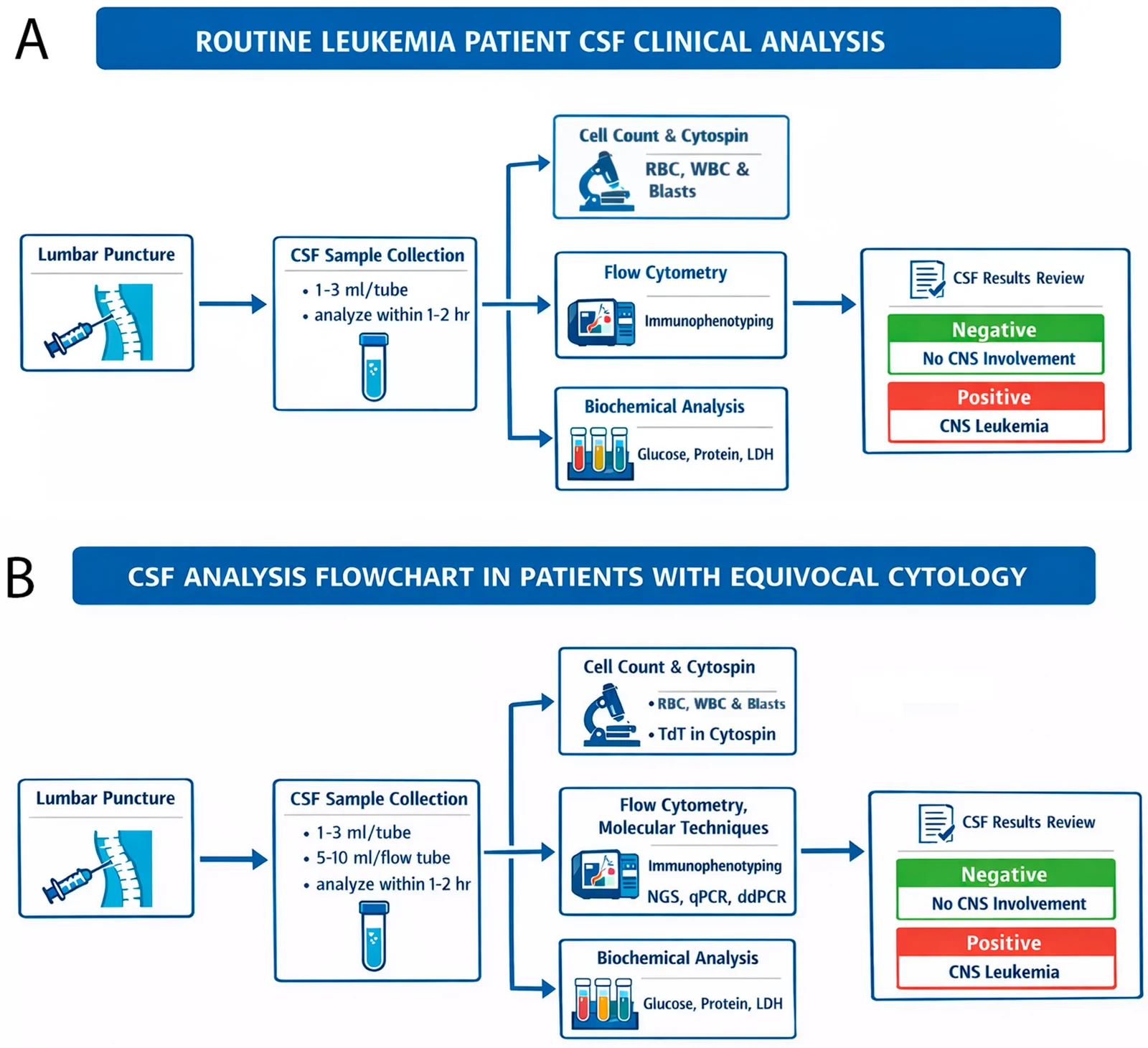
Summary of the integration of complementary diagnostic modalities to enhance the precision of CNS disease detection and support more accurate risk-adapted therapy in pediatric ALL. (**A**) Routine condition for CSF clinical analysis. (**B**) CSF analysis in patients with equivocal cytology.

CNS evaluation is critical in childhood ALL, as even low-level leukemic involvement in CSF is associated with an increased risk of relapse and inferior outcomes. Although cytomorphology providing rapid structural assessment remains the traditional diagnostic standard, its sensitivity is limited by low cellularity and subjective interpretation. Adjunct techniques, including immunocytochemistry and flow cytometry, improve diagnostic accuracy, with flow cytometry providing substantially higher sensitivity for the detection of rare leukemic blasts in routine conditions (Figure 2A). Emerging molecular approaches—such as PCR-based assays, ddPCR incorporating biomarker miRNA, and NGS for leukemia-specific genomic rearrangements—provide even greater sensitivity and hold promise for further refinement of CNS risk stratification in equivocal cytology cases (Figure 2B). Collectively, the integration of complementary diagnostic modalities enhances the precision of CNS disease detection and supports more accurate risk-adapted therapy in pediatric ALL.

## Figures and Tables

**Table 1:** Central nervous system (CNS) leukemia status criteria are primarily determined by morphological evaluation of cytospin preparations. Some institutions also recommend TdT staining and deep sequencing when patients with <5 white blood cells/µL of CSF with morphologically identifiable blasts. CNS, central nervous system. CSF, cerebrospinal fluid. N/A, not applicable. RBCs, red blood cells. TLP, traumatic lumbar puncture. WBCs, white blood cells. +, positive. −, negative. (**A**) CNS leukemia classification system used by European group and St. Jude Children’s Research Hospital. (This table was adapted with permission from an open access article: Thastrup M et al., *Leukemia*, 2022, DOI: 10.1038/s41375-022-01714-x, © Springer Nature [9].) (**B**) CNS leukemia classification system used by Children’s Oncology Group. (Table was reproduced with permission from Winick N et al., *J. Clin. Oncol.*, 2017, DOI: 10.1200/JCO.2016.71.4774, © Wolters Kluwer Health, Inc. [10] and per COG leukemia protocols.)

**(A)**			
**CNS Status**	**CSF Cytospin Findings**		
	**WBCs/µL**	**RBCs/µL**	**Leukemic Blasts**
CNS1	≤5	<10	Absent
CNS2	≤5	<10	Present
CNS3	>5	<10	Present
TLP+	N/A	≥10	Present
TLP−	N/A	≥10	Absent
**(B)**			
**CNS status**	**CSF Cytospin Findings**		
	**RBCs/µL**	**WBCs/µL**	**Leukemic Blasts**
CNS1	regardless	regardless	Negative
CNS2			
a	<10	<5	Positive for blasts
b	≥10	<5	Positive for blasts
c *	≥10	≥5	Positive for blasts, but negative by Steinherz/Bleyer algorithm
CNS3			
a	<10	≥5	Positive for blasts
b *	≥10	≥5	Positive by Steinherz/Bleyer algorithm
c	Clinical signs of CNS leukemia, such as facial nerve palsy, brain/eye involvement or hypothalamic syndrome.		

* If the patient has leukemic cells in the peripheral blood and the lumbar puncture is traumatic and contains ≥5 WBCs/µL and blasts, the following Steinherz/Bleyer algorithm is used to distinguish between CNS2 and CNS3 disease: $\frac{CSF\;WBC}{CSF\;RBC}$ > 2 × $\frac{Blood\;WBC}{Blood\;RBC}$ .

**Table 2:** Crespo-Solis’s summarization in 2012 comparing cytology versus flow cytometry analysis in patients with central nervous system malignancies [19]. CSF: cerebrospinal fluid, CNS: central nervous system. (Table was used with permission from Crespo-Solis E et al., *Curr. Oncol. Rep.*, 2012, DOI: 10.1007/s11912-012-0248-6, © Springer Science [19].)

Author/Year	Reference No.	CSF Sample Number/Type	Concordant Results
Subira, D/2001	[45]	168/Acute leukemia	94%
Roma, AA/2002	[46]	53/Lymphoma, leukemia, CNS tumor, carcinoma metastases	78.4%
Bromberg, JEC/2007	[30]	1054/Lymphoma, acute leukemia, chronic myeloid leukemia	75.8%
Sayed, D/2009	[47]	45/Acute leukemia	37.5%
Wu, JM/2009	[48]	16/Lymphoma, acute leukemia, chronic lymphocytic leukemia	52%

**Table 3:** Advantages and limitations of leukemia MRD detection methods compared with cytomorphology and flow cytometry for CSF leukemia detection. TAT, turnaround time.

Method	Target	TAT	Sensitivity	Advantage	Disadvantage
Cytomorphology	Blasts by morphology or immunochemistry staining	<24–48 h	5 × 10^−2^ (5%)	Gold standard, clear guideline available	Subjective, low sensitivity, limited reproducibility
FISH	Leukemia associated fusion genes	24–48 h	1–5 × 10^−2^ (1–5%)	Specificity	Insensitive, limited patients with aberrant karyotype (50%)
Flow cytometry	Leukemia-associated immunophenotypes	<24 h	10^−3^–10^−4^	Objective, sensitivity and specificity, short TAT	High expertise needed, optimal sample condition, machine setup
qPCR: molecular aberrations	Leukemia-specific molecular markers	48–72 h	10^−4^–10^−5^	High sensitivity and specificity, existing standardization efforts	Restricted applicability to specific target (30–50%)
ddPCR	*Ig*/*TCR* gene rearrangements, miRNA	3–5 days	10^−4^–10^−5^	High sensitivity, good applicability, no need of standard curve	Lack of standardized guidelines, higher cost, limited laboratory availability
NGS	*Ig*/*TCR* gene rearrangements	10–15 days	10^−6^	High sensitivity, high applicability, comprehensive detection, single-nucleotide resolution	Not widely available, limited standardization, need of bioinformatic analysis

## Data Availability

No new data were generated, with the exception of several images in Figure 1.

## Abbreviations

The following abbreviations are used in this manuscript:

ALL	Acute lymphoblastic leukemia
AML	Acute myeloid leukemia
cfDNA	Cell-free DNA
CNS	Central nervous system
CSF	Cerebrospinal fluid
ddPCR	Digital droplet polymerase chain reaction
FISH	Fluorescent in situ hybridization
Ig	Immunoglobulin
IgH	Immunoglobulin heavy chain
miRNA	MicroRNA
MRD	Minimal residual disease
NCCN	National Comprehensive Cancer Network
NGS	Next generation sequencing
PCR	Polymerase chain reaction
qPCR	Quantitative (Real-time) polymerase chain reaction
RBC	Red blood cell
TCR	T-cell receptor
TAT	Turnaround time
TdT	Terminal deoxynucleotidyl transferase
WBC	White blood cell
